# The roles of lipids and nucleic acids in HIV-1 assembly

**DOI:** 10.3389/fmicb.2014.00253

**Published:** 2014-05-28

**Authors:** Ayna Alfadhli, Eric Barklis

**Affiliations:** Department of Molecular Microbiology and Immunology, Oregon Health & Sciences UniversityPortland, OR, USA

**Keywords:** matrix, RNA, PI(4.5)P2, lipid, Gag

## Abstract

During HIV-1 assembly, precursor Gag (PrGag) proteins are delivered to plasma membrane (PM) assembly sites, where they are triggered to oligomerize and bud from cells as immature virus particles. The delivery and triggering processes are coordinated by the PrGag matrix (MA) and nucleocapsid (NC) domains. Targeting of PrGag proteins to membranes enriched in cholesterol and phosphatidylinositol-4,5-bisphosphate (PI[4,5]P2) is mediated by the MA domain, which also has been shown to bind both RNA and DNA. Evidence suggests that the nucleic-acid-binding function of MA serves to inhibit PrGag binding to inappropriate intracellular membranes, prior to delivery to the PM. At the PM, MA domains putatively trade RNA ligands for PI(4,5)P2 ligands, fostering high-affinity membrane binding. Triggering of oligomerization, budding, and virus particle release results when NC domains on adjacent PrGag proteins bind to viral RNA, leading to capsid (CA) domain oligomerization. This process leads to the assembly of immature virus shells in which hexamers of membrane-bound MA trimers appear to organize above interlinked CA hexamers. Here, we review the functions of retroviral MA proteins, with an emphasis on the nucleic-acid-binding capability of the HIV-1 MA protein, and its effects on membrane binding.

## INTRODUCTION

### FUNCTIONS OF RETROVIRAL MA PROTEINS

Retroviruses such as the human immunodeficiency virus (HIV) are membrane-enveloped viruses that bud from the plasma membranes of infected host cells (Coffin et al., 1997; Swanstrom and Wills, 1997; Freed, 1998; Goff, 2001). Retroviral genomes encode PrGag polyproteins that are necessary and sufficient for assembly and release of virus-like particles (VLP) from cells (Campbell and Vogt, 1995; Coffin et al., 1997; Swanstrom and Wills, 1997; Campbell and Rein, 1999; Gross et al., 2000; Campbell et al., 2001). The HIV-1 precursor Gag protein (PrGag) initially is synthesized on cytosolic ribosomes and becomes cotranslationally modified by the N-terminal attachment of a myristoyl group by *N*-myristoyl-transferase (Mervis et al., 1988; Bryant and Ratner, 1990; Ono and Freed, 1999, 2001; Morikawa et al., 2000; Ono et al., 2000; Tritel and Resh, 2000; Resh, 2004), although myristoylation is not universal among retroviral PrGag proteins (Erdie and Wills, 1990; Provitera et al., 2000; Dalton et al., 2005). PrGag proteins associate with the inner layer of the plasma membrane (PM), where they oligomerize, assemble, and bud off from cells as immature virions. The assembly process of retroviruses appears to be triggered by the association of PrGag proteins with viral RNA (vRNA) at the plasma membrane (Rein, 1994; Spearman et al., 1994; Muriaux et al., 2001; Jouvenet et al., 2006, 2008; Ott et al., 2009). However, several retroviruses, such as mouse mammary tumor virus (MMTV) and the Mason-Pfizer monkey virus (MPMV), assemble within the cytoplasm before being transported to cell membrane (Choi et al., 1999; Stansell et al., 2007). During the maturation process, cleavage of HIV-1 PrGag by the viral protease (PR) generates the mature myristoylated matrix (MA) protein as well as capsid (CA), nucleocapsid (NC), p6 and two spacer peptides, Sp1 and Sp2 (Swanstrom and Wills, 1997; Freed, 1998).

The MA domain plays multiple roles in the viral replication cycle. One of these roles involves the incorporation of the viral envelope (Env) proteins into virus particles. Evidence indicates that HIV-1 MA interacts with the cytoplasmic tail (CT) of gp41, the transmembrane (TM) portion of the HIV-1 Env protein, to facilitate the incorporation of Env proteins into assembling virions (Yu et al., 1992; Dorfman et al., 1994; Freed and Martin, 1995, 1996; Wyma et al., 2000; Davis et al., 2006; Checkley et al., 2011). Several models have been proposed to explain the incorporation of retroviral Env protein into virus particles (reviewed by Checkley et al., 2011). One of these models is the passive model, in which membrane proteins at assembly sites are incorporated into virions as innocent bystanders. This model was based on the observation that retroviruses could incorporate foreign membrane proteins into their envelopes. When glycoproteins from heterologous viruses are assembled into a retrovirus envelope, the process is termed pseudotyping (Zavada, 1982; Lusso et al., 1990; Arthur et al., 1992; Ott, 2008; Checkley et al., 2011). For example, infectious HIV-1 particles can be produced with foreign glycoproteins such as the vesicular stomatitis virus G protein (VSV-G; ) or amphotropic murine leukemia virus (MLV) Env (). In these cases, HIV-1 cores and genomes are delivered to target cells carrying the VSV or MLV receptors (Jorgenson et al., 2009). The passive model also is supported by the finding that removal of most of HIV-1 gp41 CT has a moderate effect on Env glycoprotein incorporation into HIV-1 particles (Wilk et al., 1992; Freed and Martin, 1995, 1996; Akari et al., 2000; Murakami and Freed, 2000).

Although the passive model is consistent with observations for a number of retroviruses (Landau et al., 1991; Reiser et al., 1996; Lewis et al., 2001; Liu et al., 2004; Jorgenson et al., 2009), several lines of evidence suggest an interaction between MA and Env. For HIV-1, it has been reported that mutations in MA may decrease levels of HIV-1 Env incorporation into virions (Wang et al., 1993; Freed and Martin, 1995, 1996; Reil et al., 1998). Moreover, some mutations in MA (Freed and Martin, 1995, 1996; Reil et al., 1998) have been shown to mitigate the effects of certain Env mutations. Interestingly, for some but not all cell types, MA mutations can be compensated via full deletions of the HIV-1 Env protein cytoplasmic tail (CT; Freed and Martin, 1995, 1996; Checkley et al., 2011). These results suggest that while truncated HIV-1 Env can be incorporated passively into virions in some cell types, full-length Env requires an interaction with MA for assembly into virions (Freed and Martin, 1995, 1996; Checkley et al., 2011). Data from other experiments indicate that MA domains in immature PrGag lattices lock Env proteins into a non-fusogenic state, and that PrGag processing serves as a switch to regulate envelope protein function (Murakami et al., 2004; Wyma et al., 2004; Jiang and Aiken, 2007). *In vitro* studies have shown direct binding between MA and the CT Env in several biochemical experiments for both HIV-1 and Simian immunodeficiency virus (SIV; Cosson, 1996; Wyma et al., 2000; Manrique et al., 2008). Consistent with these observations, structural studies have shown that HIV-1 MA proteins assemble lattices on phosphatidylinositol-(4,5)-bisphosphate (PI[4,5]P2) membranes in which residues implicated in CT binding point toward lattice holes (Yu et al., 1992; Dorfman et al., 1994; Freed and Martin, 1996; Ono et al., 1997; Murakami and Freed, 2000; Davis et al., 2006; Bhatia et al., 2007; Alfadhli et al., 2009a; Checkley et al., 2011; Tedbury et al., 2013). Given this membrane organization of MA, it seems likely that membrane proteins with short cytoplasmic domains may enter Gag lattices passively, whereas proteins such as HIV-1 Env, with long cytoplasmic tails require MA interactions.

Implicit in the data described above is the assumption that MA binds to membranes, and another essential function of MA is to target PrGag proteins to their lipid raft assembly sites at the PMs of infected cells (Ehrlich et al., 1996; Spearman et al., 1997; Scarlata et al., 1998; Bouamr et al., 2003; Murray et al., 2005; Jouvenet et al., 2006; Bhatia et al., 2007; Dalton et al., 2007; Scholz et al., 2008; Hamard-Peron and Muriaux, 2011). In most mammalian retroviruses, membrane targeting is dependent on two structural features present on MA: the N-terminal myristyl group and a group of basic residues. For such viruses, the N-terminal myristyl group functions in concert with a group of conserved basic residues to promote membrane binding (Zhou et al., 1994; Tang et al., 2004; Saad et al., 2008). However, Gag proteins of some retroviruses, such as Rous sarcoma virus (RSV) and equine infectious anemia virus (EIAV), lack the myristate anchor, and Gag targeting and binding to the PM is mainly mediated by electrostatic interactions (Erdie and Wills, 1990; Parent et al., 1996; Callahan and Wills, 2000; Provitera et al., 2000; Dalton et al., 2005). Compelling evidence favors the idea that HIV assembly does not occur just anywhere at the PM, but at lipid rafts and at PI(4,5)P2-enriched areas (Ono et al., 2004; Chukkapalli et al., 2008, 2010; Chukkapalli and Ono, 2011). MA–PI(4,5)P2 interactions also have been observed for MLV, MPMV, and EIAV (Stansell et al., 2007; Chan et al., 2008; Chen et al., 2008; Hamard-Peron et al., 2010). In cell culture, decreasing the levels of cellular PI(4,5)P2 by overexpression of polyphosphoinositide 5-phosphatase IV was shown to reduce HIV-1 and MLV assembly efficiency, resulting in the delivery of viral proteins to intracellular compartments (Ono et al., 2004; Chan et al., 2008; Chukkapalli et al., 2008; Hamard-Peron et al., 2010; Inlora et al., 2011). In contrast, recent studies have shown that human T-lymphotropic virus type 1 (HTLV-1) Gag is markedly less dependent on PI(4,5)P2 for membrane binding and particle release than HIV-1 Gag (Inlora et al., 2011). For RSV, Chan et al. (2011) reported that RSV Gag bound effectively to a variety of phosphorylated phosphatidylinositols, and that reduction of cellular PI(4,5)P2 and PI(3,4,5)P3 levels did not reduce Gag PM binding or virus particle release. However, more recently, Nadaraia-Hoke et al. (2013) reported that depletion of cellular PI(4,5)P2 and PI(3,4,5)P3 yielded reductions of both RSV Gag PM binding and virus particle release. Interestingly, RSV Gag mutants that are impaired in nuclear trafficking were less sensitive to these effects, suggesting a link between RSV Gag PM targeting and nuclear trafficking (Nadaraia-Hoke et al., 2013).

In addition to Env protein and membrane binding, several reports have implicated nucleic acid binding properties to retroviral MAs. These viruses are HIV-1 (Luban and Goff, 1991; Bukrinsky et al., 1993; Von Schwedler et al., 1994;; Lochrie et al., 1997; Miller et al., 1997; Reil et al., 1998; Haffar et al., 2000; Purohit et al., 2001; Ott et al., 2005; Hearps et al., 2008; Alfadhli et al., 2009b, 2011; Cai et al., 2010; Chukkapalli et al., 2010, 2013; Monde et al., 2011), RSV (Leis et al., 1978, 1980; Steeg and Vogt, 1990), and BLV (Mansky et al., 1995; Mansky and Wisniewski, 1998; Mansky and Gajary, 2002; Wang et al., 2003). While the NC domains of retroviral PrGag proteins are essential for viral RNA (vRNA) encapsidation, experiments have shown that MA proteins may also possess binding functions and can substitute for the HIV-1 NC protein assembly function (Ott et al., 2005). [However, deletion of the NC domain dramatically reduces the assembly of MLV particles (Muriaux et al., 2004)]. It has been conjectured that such MA–nucleic acid binding might facilitate PrGag delivery to the PM, virus assembly, and/or nuclear import of viral preintegration complexes (PICs) (Bukrinsky et al., 1993; Von Schwedler et al., 1994; Miller et al., 1997; Reil et al., 1998; Haffar et al., 2000; Hearps et al., 2008; Cai et al., 2010). In this review, we focus on the role of MA binding to RNA and summarize the importance of Gag MA interactions with RNA for HIV and other retroviruses, with the hope that this comparative approach can shed more light on our understanding of the importance of this function and ways of inhibiting that role.

## STRUCTURAL ASPECTS OF RETROVIRAL MATRIX PROTEINS

**FIGURE 1:**
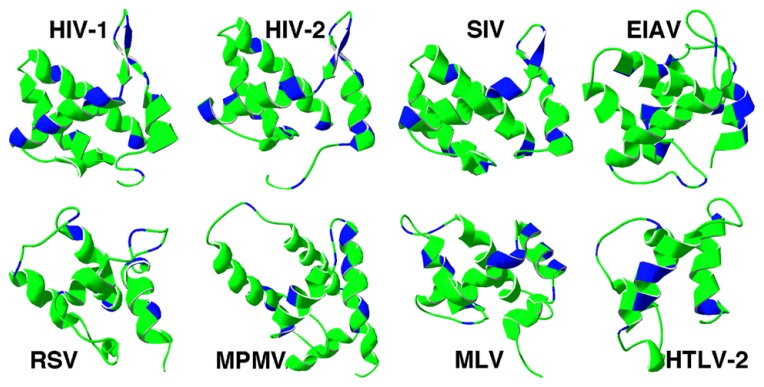
**Retrovirus matrix protein membrane binding regions.** Ribbon diagrams of the membrane binding regions of four lentivirus matrix proteins (top row) and an alpharetrovirus (RSV), betaretrovirus (MPMV), gammaretrovirus (MLV), and deltaretrovirus (HTLV-2) are depicted. In each case, matrix helix one is on the right-hand side of the figure, and basic residues are indicated in blue. The PDB files for each matrix protein are as follows: HIV-1 (1UPH), HIV-2 (2K4H), SIV (1ECW), EIAV (1HEK), RSV (1A6S), MPMV (1BAX), MLV (1MN8), HTLV-2 (1JVR).

Matrix structures for the following retroviruses have been determined: HIV- 1 (Massiah et al., 1994; Hill et al., 1996; Tang et al., 2004; Saad et al., 2006, 2007), HIV-2 (Saad et al., 2008), SIV (Rao et al., 1995), human T-lymphotropic virus 2 (HTLV-2; Christensen et al., 1996), BLV (Matthews et al., 1996), M-PMV (Conte et al., 1997), Rous sarcoma virus (RSV; N-terminal fragment; McDonnell et al., 1998), EIAV (Hatanaka et al., 2002), and MLV (Riffel et al., 2002). In contrast to their low sequence homology, structures of retroviral MA proteins are remarkably similar (**Figure 1**; Murray et al., 2005). They all share a globular core composed of α helices. The N-termini of the MA proteins tend to contain basic residues that appear to foster interactions of MA with acidic phospholipid head groups. Another essential element that contributes to membrane binding is the myristyl group found in most retroviral MAs, including HIV-1 (Gottlinger et al., 1989; Bryant and Ratner, 1990; Spearman et al., 1994), HIV-2 (Saad et al., 2008), MLV (Henderson et al., 1983), M-PMV (Schultz and Oroszlan, 1983), and HTLV (Ootsuyama et al., 1985). However, there are exceptions, such as RSV and EIAV viruses (Schultz and Oroszlan, 1983), which do not have myristoylated MA proteins.

A number of structural studies have been conducted on HIV-1 MA (Massiah et al., 1994; Hill et al., 1996; Tang et al., 2004; Saad et al., 2006, 2007; Alfadhli et al., 2009a). In addition to its N-terminal myristate, which is essential for efficient membrane binding (Gottlinger et al., 1989; Bryant and Ratner, 1990; Freed et al., 1994; Spearman et al., 1994; Tang et al., 2004; Saad et al., 2006, 2007), the HIV-1 MA protein is composed of six α helices and three β sheet strands (Massiah et al., 1994; Hill et al., 1996; Tang et al., 2004; Saad et al., 2006, 2007). Sedimentation equilibrium data have shown that while myristoylated HIV-1 MA exists in a monomeric–trimeric state at equilibrium, unmyristoylated MA occurs as a monomer even at high concentrations (Tang et al., 2004). NMR studies suggest that upon Gag multimerization the myristoyl group is exposed, and this fosters Gag binding to membranes (Tang et al., 2004; Saad et al., 2006, 2007).

**FIGURE 2:**
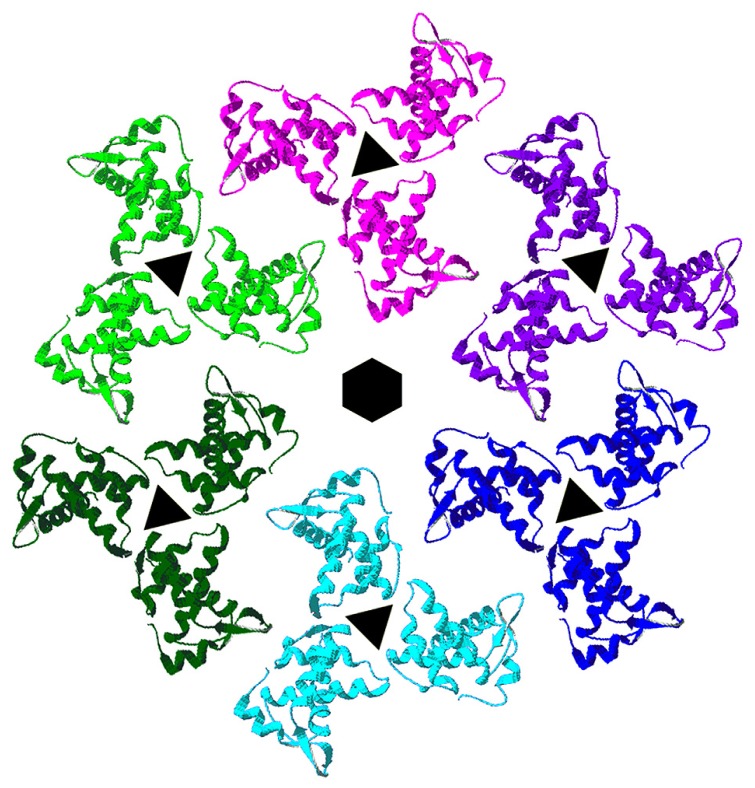
**Membrane organization of HIV-1 matrix proteins.** Trimers of HIV-1 matrix proteins (PDB 1HIW) were fitted onto the electron densities of HIV-1 MA proteins assembled onto a lipid monolayer containing PI(4,5)P2 (Alfadhli et al., 2009a). As shown, trimers organize in hexameric rings around protein-free holes. MA residues that have been shown to affect HIV-1 Env protein assembly into virions locate toward the centers of the hexameric holes, implying that the cytoplasmic tails of HIV-1 Env proteins occupy these spaces.

The membrane binding face of HIV-1 MA is basic, promoting interactions with negatively charged phospholipid head groups at the inner leaflets of PMs (Massiah et al., 1994; Zhou et al., 1994; Hill et al., 1996; Tang et al., 2004; Saad et al., 2006, 2007). NMR investigations have indicated that HIV-1 MA preferentially binds to soluble PI(4,5)P2 mimics through contacts with the lipid head group and its 2′ acyl chain, and that binding promotes both exposure of the MA myristate group and protein oligomerization (Tang et al., 2004; Saad et al., 2006, 2007). Consistent with the above observations, it has been shown that HIV-1 MA and MACA proteins tend to organize as hexamers of trimers on lipid membranes containing PI(4,5)P2 (**Figure 2**; Alfadhli et al., 2009a), and that the binding specificity of MA is enhanced by cholesterol (Alfadhli et al., 2009a, b). These results suggest a model in which each MA trimer contributes to three separate hexamer rings, and MA proteins are positioned roughly above CA N-terminal domain (NTD) hexamers, which also are linked via CA C-terminal domain (CTD) contacts. This model implies that the shells of immature HIV-1 virions are stabilized by multiple Gag domain contacts, and has implications for how Env proteins assemble and fit into virus particles. Significantly, MA residues shown to be critical for incorporation of HIV-1 Env proteins orient toward the hexameric holes in the lattices (Yu et al., 1992; Dorfman et al., 1994; Freed and Martin, 1996; Ono et al., 1997; Murakami and Freed, 2000; Davis et al., 2006; Bhatia et al., 2007; Alfadhli et al., 2009a; Checkley et al., 2011; Tedbury et al., 2013).

## RETROVIRAL MA BINDING TO NUCLEIC ACIDS

For a number of years, researchers have reported that retroviral MA proteins possess nucleic-acid-binding properties (Sen and Todaro, 1977; Leis et al., 1978, 1980; Steeg and Vogt, 1990; Katoh et al., 1991, 1993; Luban and Goff, 1991; Bukrinsky et al., 1993; Von Schwedler et al., 1994; Mansky et al., 1995; Lochrie et al., 1997; Miller et al., 1997; Swanstrom and Wills, 1997; Mansky and Wisniewski, 1998; Reil et al., 1998; Haffar et al., 2000; Garbitt et al., 2001; Purohit et al., 2001; Mansky and Gajary, 2002; Wang et al., 2003; Hearps et al., 2008; Alfadhli et al., 2009b, 2011; Cai et al., 2010; Chukkapalli et al., 2010, 2013; Chukkapalli and Ono, 2011; Monde et al., 2011). In early studies on retroviruses such as avian sarcoma and leukemia viruses (ASLV) and RSV, which is closely related to ASLV, MA was reported to be associated with vRNA in virus particles (Sen and Todaro, 1977; Leis et al., 1978, 1980), although subsequent work attributed this activity to NC (Meric et al., 1984). In any event, the binding of RSV MA to RNA is not of high specificity (Steeg and Vogt, 1990), and studies have shown that RSV MA binds to vRNA, ribosomal RNA, and DNA with similar affinities (Meric and Spahr, 1986; Parent and Gudleski, 2011).

Early studies using RNA gel mobility shift assays, and radioactive cDNA hybridization and mapping studies implicated the MA domain of BLV Gag in specific binding of vRNA (Katoh et al., 1991, 1993). Although the BLV Gag NC domain contains two zinc finger domains and basic amino acids that are important for vRNA packaging (Wang et al., 2003), the mature BLV NC proteins lack selectivity for vRNA sequences containing the encapsidation signal (Katoh et al., 1991, 1993). Surprisingly, the BLV precursor MA(p15) protein binds specifically to two distinct regions of viral RNA (Mansky and Wisniewski, 1998). This observation is discussed in more detail below.

For HIV-1, *in vitro* selection experiments identified RNA aptamers that showed high-affinity binding to HIV-1 MA (Lochrie et al., 1997; Purohit et al., 2001; Ramalingam et al., 2011). Lochrie et al. (1997) identified RNA ligands that bind to two different regions within Gag, either to MA or to NC. These RNAs were 50-mer aptamers and had dissociation constants between 3 and30 nM (Lochrie et al., 1997). However, the RNA sequences identified by this screen were not related to any region on the HIV-1 vRNA (Lochrie et al., 1997). Subsequently, Purohit et al. (2001) identified high-affinity RNA ligands to HIV-1 MA that were selected by screening of random 76-mer and 31-mer RNA libraries. These investigators showed that MA binds directly to an RNA sequence that is homologous to a fragment of the *pol* sequence with an affinity of about 500 nM (Purohit et al., 2001; Alfadhli et al., 2011). The region of MA that binds to this RNA was restricted to the N-terminal basic domain, and substitution in the basic residues led to weak binding to RNA *in vitro*. Viral mutants that interfered with the MA–RNA interaction yielded a 4–5 day replication delay *in vivo* (Purohit et al., 2001). However, it is possible that mutations that affected RNA binding also affected other viral functions. In the third study (Ramalingam et al., 2011), MA-binding aptamers were found with *K*_d_s in the range of 100–250 nM, but expression of these aptamers in cells had only minimal effects on HIV-1 functions.

**FIGURE 3:**
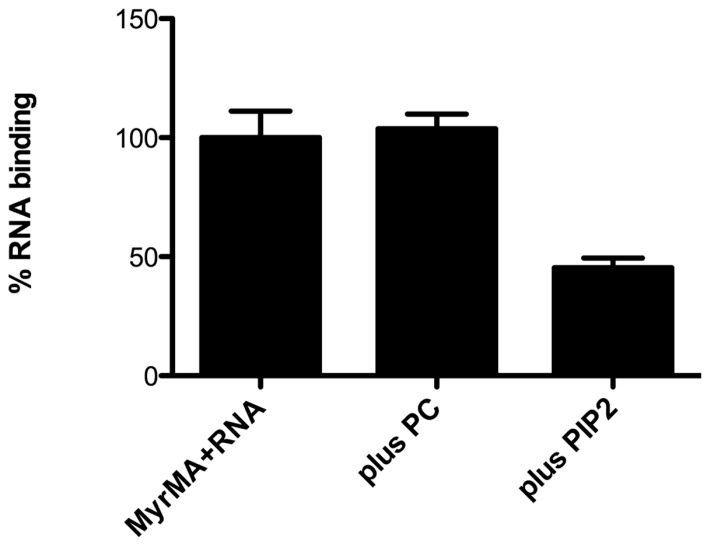
**Competition of PI(4,5)P2 liposomes for matrix protein RNA binding sites.** Experiments performed by Alfadhli et al. (2011) have shown that nickel-nitrilotriacetic acid (Ni-NTA) beads coated with the myristoylated HIV-1 matrix protein (MyrMA) bind fluorescently tagged Sel25 RNA (MyrMA + RNA). Addition of liposomes composed of 80% dioleoylphosphatidyl choline + 20% cholesterol (plus PC) during binding reactions did not diminish MA–RNA binding, whereas addition of an equal amount of liposomes composed of 70% PC, 10% PI(4,5)P2, 20% cholesterol (plus PIP2) significantly reduced MA–RNA binding. These and other experiments indicate that the affinity of HIV-1 MA for RNA is less than that for membranes containing PI(4,5)P2 but greater than that for membranes without PI(4,5)P2.

Recent studies with HIV-1 MA provide corroboration of its RNA-binding capacity. In particular, bead binding experiments have indicated that fluorescently tagged RNAs and DNAs bind well to HIV-1 MA but not to control proteins (Alfadhli et al., 2009b). Interestingly, it has been shown that RNA binding enhances the binding specificity of MA to PI(4,5)P2-containing membranes. This was indicated by the fact that PI(4,5)P2-containing liposomes successfully competed with nucleic acids for MA binding, whereas other liposomes did not (**Figure 3**; Alfadhli et al., 2009b). In agreement with these results, other studies have shown that the highly basic region (HBR) on the N-terminal portion of MA not only contributes to binding of PI(4,5)P2, but also is capable of binding to RNA (Chukkapalli et al., 2008, 2010, 2013). Furthermore, RNAse treatment of *in vitro* translated Gag protein preparations decreased the binding specificity to membranes containing PI(4,5)P2, suggesting that RNA influences the membrane binding specificity of MA (Chukkapalli et al., 2008, 2010, 2013, see below).

In support of these studies, it has been shown by Burniston et al. (1999) that the basic residues of the HIV-1 MA domain contribute to Gag–Gag interactions in the presence of RNA and the absence of the NC domain, indicating that the basic residues on MA play a role in RNA binding (Burniston et al., 1999). NMR studies also have confirmed interactions of HIV-1 MA with RNA and DNA, and have implicated the nucleic-binding surfaces on MA (Cai et al., 2010; Alfadhli et al., 2011). As discussed above, over a decade ago *in vitro* selection experiments identified a 25-mer aptamer that showed high-affinity binding to HIV-1 MA (Purohit et al., 2001). More recently, MA binding to this aptamer has been characterized. MA–RNA binding was verified via gel shift assays, fluorescence anisotropy (FA) assays, analytical ultracentrifugation, and NMR methods (Alfadhli et al., 2011).

In summary, numerous studies have shown that the MA domains from different retroviruses possess nucleic-acid-binding properties. The significance of these interactions and their plausible roles are described below.

## THE ROLE OF MA–NUCLEIC ACID BINDING

### THE ROLE OF MA IN RNA ENCAPSIDATION

The RNA encapsidation process for retroviruses involves recognition of the vRNA by the viral Gag polyprotein, and it is essential for the assembly of infectious virions. Biochemical and genetic studies have revealed that encapsidation involves the association between a stable RNA structure near the 5′ ends of viral genome called the encapsidation (Ψ, packaging) signal and, in most cases, amino acid residues in the NC domain of the Gag protein (Mansky et al., 1995; Berkowitz et al., 1996; Swanstrom and Wills, 1997; Parent and Gudleski, 2011). It has been shown that RSV NC is essential for efficient vRNA encapsidation (Dupraz and Spahr, 1992; Aronoff et al., 1993; Lee et al., 2003; Lee and Linial, 2004; Zhou et al., 2005). However, studies have shown that other regions of RSV PrGag contribute to RNA packaging (Sakalian et al., 1994; Parent and Gudleski, 2011). In particular, mutations of the N-terminal region of RSV MA have resulted in defects in RNA dimerization and encapsidation (Sakalian et al., 1994; Garbitt-Hirst et al., 2009). The RSV Gag proteins are synthesized in the cytosol, and were believed that to be targeted directly to the plasma membrane. However, genetic and biochemical studies have indicated that RSV MA and NC domains contain nuclear localization signals (NLS) for nuclear targeting (Butterfield-Gerson et al., 2006; Garbitt-Hirst et al., 2009; Gudleski et al., 2010; Baluyot et al., 2012). Studies by Parent and co-workers have indicated that RSV MA influences vRNA encapsidation indirectly, and have proposed a working model for RSV MA role in vRNA packaging (Scheifele et al., 2002; Butterfield-Gerson et al., 2006; Garbitt-Hirst et al., 2009; Gudleski et al., 2010). According to this model, the NLS on MA binds directly to importin-11 and/or the NC NLS binds to the importin-alpha/importin-beta complex, and Gag nuclear import is directed by the importins. Once in the nucleus, Gag is released from import factors and binds to vRNA, primarily through an interaction of the NC domain with the packaging signal. RSV Gag–RNA binding may induce conformational changes in RSV Gag that expose a nuclear export signal (NES) in the Gag p10 domain (Garbitt-Hirst et al., 2009; Gudleski et al., 2010). This proposed conformational change appears to promote binding of the Gag p10 NES directly to CRM-1/RanGTP, a major exporter of RNA-binding proteins from the nucleus (Scheifele et al., 2002; Garbitt-Hirst et al., 2009; Gudleski et al., 2010). The Gag–RNA complex is then exported through the nuclear pore and travels to the plasma membrane where Gag undergoes multimerization and budding. However, it should be noted that a chimeric protein with the HIV-1 MA domain fused to the remainder of RSV Gag was able to replicate in a single round infectivity assay even though nuclear trafficking of the HIV/RSV chimeric protein was not readily detected by fluorescence microscopy (Baluyot et al., 2012).

In contrast to RSV, the BLV MA appears to play a direct role in vRNA encapsidation. While the NC domain of BLV plays a major role in genome recognition and RNA encapsidation, evidence in the literature implicates the MA protein of BLV in these events (Katoh et al., 1991, 1993; Mansky and Wisniewski, 1998). In particular, the MA domain of BLV PrGag is involved in the specific selection and packaging of vRNAs (Katoh et al., 1991, 1993; Parent and Gudleski, 2011). As one line of evidence, RNA gel mobility shift assays have shown that BLV MA binds specifically to RNAs representing the 5′ region of the BLV vRNA (Katoh et al., 1991, 1993). Furthermore, cDNA hybridization and mapping studies demonstrated that the BLV MA specifically binds to two different segments of the vRNA (Katoh et al., 1991, 1993). The first RNA region contains the vRNA dimerization domain, while the second RNA region is at the 5′ end of the *gag* gene, which is the location of the encapsidation signal for BLV (Mansky and Wisniewski, 1998). Interestingly, it is the BLV precursor MA (p15) protein and not the mature MA (p10) that binds specifically to the vRNA dimerization element and the encapsidation signal (Mansky and Wisniewski, 1998). Moreover, studies conducted by Katoh et al. (1991) showed that the BLV NC protein possesses only a non-specific RNA-binding activity, with little selectivity for the vRNA encapsidation signal. However, studies conducted by Mansky and co-workers provide genetic evidence that both the MA and NC domains of BLV PrGag are needed for efficient RNA packaging (Mansky et al., 1995; Mansky and Wisniewski, 1998; Mansky and Gajary, 2002; Wang et al., 2003). Mutational analysis of MA and NC showed that charged residues within both of these regions of Gag are needed for optimal genome packaging (Wang et al., 2003). In particular, mutation of residues K41 and H45 in MA, and of basic and zinc finger residues on NC resulted in BLV vRNA encapsidation defects (Wang et al., 2003). Thus, BLV provides one example in which the MA–RNA binding function is directly employed in the viral replication strategy.

### THE ROLE OF HIV-1 MA IN NUCLEAR IMPORT

Historically, HIV-1 MA was the first protein implicated in directing the nuclear import of pre-integration complexes (PICs) early in infection (Bukrinsky et al., 1993; Von Schwedler et al., 1994). Reports indicated that HIV-1 MA contains an NLS that maps to the basic residues 25–33 (Bukrinsky et al., 1993; Von Schwedler et al., 1994; Depienne et al., 2000), and that mature MA enters infected cells along with vRNA and other viral proteins. Moreover, some MA molecules were found to be localized to PICs (Bukrinsky et al., 1993; Von Schwedler et al., 1994), and it thus was originally proposed that the MA NLS facilitates nuclear translocation of PICs prior to proviral integration (Bukrinsky et al., 1993; Von Schwedler et al., 1994; Miller et al., 1997; Reil et al., 1998; Haffar et al., 2000).

However, a number of reports challenged the role of HIV-1 MA in directing the nuclear import of PICs (Freed et al., 1994; Fouchier et al., 1997; Reil et al., 1998; Hearps et al., 2008). Notably, Gottlinger and co-workers showed that viruses lacking most of MA were capable of infecting non-dividing cells, suggesting that the putative MA NLS is not essential for HIV-1 replication (Reil et al., 1998; Depienne et al., 2000). Hearps et al. (2008) assessed the nuclear import properties of GFP-tagged MA, and concluded that MA is excluded from the nuclei of transfected cells. MA also failed to enter the nuclei of cells in *in vitro* transport assays using cells with perforated PMs but intact nuclear membranes (Hearps et al., 2008). Nevertheless, MA mutants have been shown to affect proviral DNA circularization and integration (Mannioui et al., 2005), and MA binding to DNA was demonstrated using *in vitro* DNA gel shift analysis (Hearps et al., 2008). Instead of a nuclear localization role for HIV-1 MA, these observations suggest that MA associates with PICs and augments integration. Recent NMR studies showing that MA residues R22, K27, Q28, K30, and K32 mediate binding to dsDNA (Cai et al., 2010) are consistent with this notion.

### THE ROLE OF HIV-1 MA BINDING TO RNA: REGULATION OF MEMBRANE BINDING

Studies by Alfadhli et al. (2009b, 2011) demonstrated that MA binds to nucleic acids, and that PI(4,5)P2-containing liposomes successfully compete with nucleic acids for MA binding, whereas other liposomes do not (Alfadhli et al., 2009b, 2011; **Figure 3**). Complementary studies by Ono and co-workers indicated that RNase treatment of Gag *in vitro* translation extracts reduced the selectivity of Gag binding for PI(4,5)P2 (Chukkapalli et al., 2010, 2013; Chukkapalli and Ono, 2011). These studies imply that MA and NC domains of HIV-1 PrGag bind to RNA in the cytoplasm of infected cells until PrGag reaches PI(4,5)P2-rich domains at the plasma membrane. By this scenario, MA–RNA binding increases the specificity of PrGag for PI(4,5)P2. This could be plausible if the MA affinity for RNA were between its affinity for PI(4,5)P2 and that for other phospholipids, so that RNA binding could protect MA from binding to inappropriate membranes (Alfadhli et al., 2009b, 2011; Chukkapalli et al., 2010, 2013; Chukkapalli and Ono, 2011). Consistent observations by Jones et al. (2011), showed that both MA and NC PrGag domains can bind nucleic acids, and that binding of MA to inositol phosphate (IP) derivatives, which resemble the PI(4,5)P2 head group, alters the association of PrGag to nucleic acids. Notably, experiments demonstrated that *in vitro* tRNA annealing to vRNA catalyzed by PrGag is enhanced over 10-fold by the addition of IPs to the reaction (Jones et al., 2011). In contrast, the IPs had no effect on the annealing induced by NC alone or CA–NC proteins. These results show that MA and NC can bind to nucleic acids, and that MA–RNA binding reduces tRNA annealing. By this model, IPs compete with vRNAs for MA binding, allowing NC to perform its encapsidation and annealing functions (Rein, 2010; Jones et al., 2011).

**FIGURE 4:**
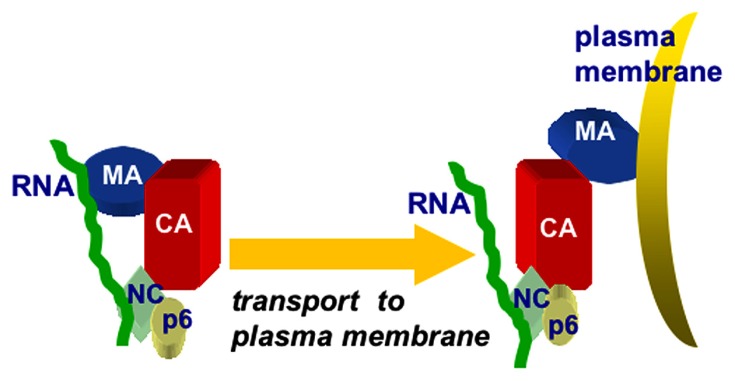
**Chaperone model for MA–RNA binding.** The chaperone model for MA-RNA binding posits that both the MA and NC domains of PrGag proteins bind to RNA in the cytoplasm of infected cells, and that the MA–RNA binding protects MA from binding to inappropriate intracellular membranes, lacking PI(4,5)P2. Once at PI(4,5)P2-rich sites at the plasma membrane, MA binding switches from RNA to PI(4,5)P2, facilitating the trafficking and assembly of PrGag proteins.

Another line of investigation based on biochemical and structural studies using hydrodynamic and small angle neutron scattering (SANS) methods showed that the Gag protein adopts a compact bent shaped conformation in solution. When only RNA is added, the Gag proteins assemble very small VLPs, suggesting that both MA and NC domains bind to RNA. However, in the presence of both RNA and IP membrane mimics, Gag undergoes a conformational switch to an extended rod-shaped form (Datta et al., 2007, 2011). Overall, the data above suggest that RNA provides a chaperone function in preventing HIV-1 Gag proteins from binding to membranes until they reach PI(4,5)P2-rich plasma membranes. Such a model is depicted in **Figure 4**, which illustrates the binding of PrGag MA and NC domains to vRNA, followed by an MA switch to membrane binding at PM assembly sites.

To test the MA–RNA chaperone model, Ono and co-workers measured cellular RNA levels and found that they are sufficient for blocking PrGag binding to phosphatidylserine (PS; Chukkapalli et al., 2013). These results provide cell-based evidence supporting the notion that RNA regulates membrane binding, and prevents PrGag from binding promiscuously to PS-containing membranes (Chukkapalli et al., 2013). However, recent studies conducted by Dick et al indicate that RNA regulation of PrGag membrane binding is not universal among retroviruses (Dick et al., 2013). In contrast to HIV-1 Gag, RNAse treatment of reticulocyte lysates containing *in vitro*-translated RSV Gag did not alter the protein’s membrane-binding characteristics. Potentially, this is because the interactions of RSV MA with RNA are weaker than those of HIV MA (Dick et al., 2013).

Given the implicated chaperone role for HIV-1 MA-nucleic acid binding (Chukkapalli et al., 2008, 2010, 2013; Alfadhli et al., 2009b, 2011), some aspects of HIV-1 MA–RNA binding have been examined further. One basic question relating to these observations concerns the nature of MA–RNA interactions. Previously, *in vitro* selection experiments identified a 25-mer RNA aptamer called Sel25 (GGACA GGAAU UAAUA GUAGC UGUCC) which demonstrated high-affinity binding to HIV-1 MA (Lochrie et al., 1997; Purohit et al., 2001). The central fifteen nucleotides (Sel15) also showed high-affinity binding to the protein. As a step toward characterization of MA–RNA interactions, MA binding to Sel25, Sel15, and their random sequence counterparts (Ran25, Ran15) was tracked via gel shift assays, fluorescence anisotropy (FA) assays, and analytical ultracentrifugation methods (Alfadhli et al., 2011). These investigations confirmed the specificity of MA binding to Sel15 and Sel25 RNAs. In addition, these studies identified RNA as a competitor for membrane binding, and assays indicated that PI(4,5)P2-containing liposomes significantly reduced RNA binding to MA. *In vitro* competition binding experiments also showed that a soluble PI(4,5)P2 mimic (PIPC8) reduced Sel25 binding to MA, whereas a soluble PS mimic did not; while FA competition data indicated that PIPC8 reduced MA–RNA binding levels to a greater extent than did the PS mimic (Alfadhli et al., 2011). These results are consistent with the notion that RNA increases the ability of MA to distinguish between phospholipid head groups.

**FIGURE 5:**
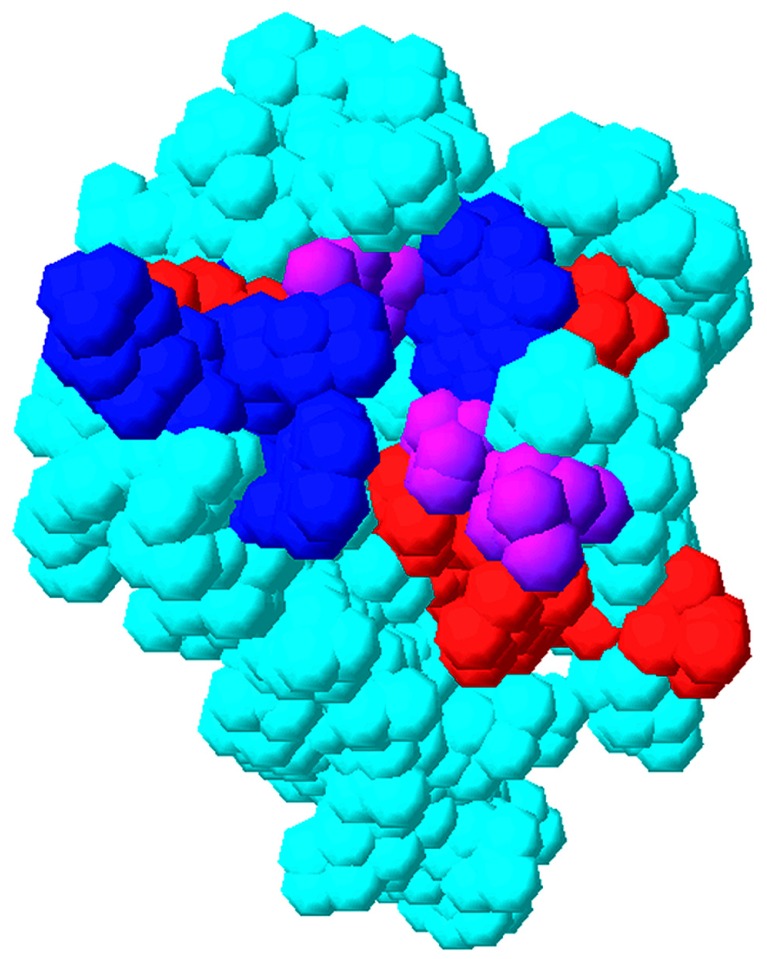
**Overlap of matrix RNA and PI(4,5)P2 binding sites.** The membrane-binding surface of the HIV-1 matrix protein (PDB 1UPH) is illustrated as a space-filling model. Residues that have been implicated in PI(4,5)P2 binding are indicated in blue, residues that have been implicated in RNA binding (Alfadhli et al., 2011) are indicated in red, and residues that have been implicated in both PI(4,5)P2 and RNA binding are indicated in purple.

What MA surfaces are sensitive to RNA binding? In an attempt to address this question, NMR binding studies were performed. Using this approach, MA residues at the putative RNA binding site were identified by their chemical shift perturbations upon titration. In particular, significant NMR shifts were observed for residues located to the matrix protein β-II-V cleft corresponding to residues Gln-28, His-33, Glu-40, Glu-42, Ile-60, Leu-68, Thr-70, Glu-73, Arg-76, Ser-77, Tyr-79, and Asn-80 (**Figure 5**; Alfadhli et al., 2011). Some of these residues (residues 33, 73, 76, and 79) previously were shown to contribute to the PI(4,5)P2 binding site (**Figure 5**; Saad et al., 2006). This observed overlap of PI(4,5)P2-MA and RNA–MA binding sites reinforces a chaperone function hypothesis. These results also are in agreement with other NMR studies which implied that MA residues 28–33 and, to a lesser extent, residues 70–79 contribute to MA–DNA binding in preintegration complexes (Cai et al., 2010). It also is pertinent to note that NMR titrations indicated residues 94, 97, 103, and 104 were affected by RNA titrations (Alfadhli et al., 2011). These residues are located on MA helix VI and may involve a conformational change of MA upon RNA binding that also could affect binding specificity. While the sum of the above results support a hypothesis in which MA–RNA binding is utilized by HIV-1 to regulate virus assembly, the identity of the RNA(s) that bind to MA *in vivo* remains to be determined. In this regard, it is noteworthy that a nearly exact match of the Sel15 RNA sequence is located in the *pol* coding region of HIV-1, but while mutations of consensus nucleotides involved in MA–Sel RNA binding reduced binding *in vitro*, they only modestly reduced viral infectivity *in vivo* (Purohit et al., 2001). Thus, it is likely that MA can bind to other sequences on viral or cellular RNAs to effect its chaperone functions.

## INHIBITION OF HIV-1 MA/RNA BINDING

Despite the effectiveness of the current highly active antiretroviral therapy (HAART) in the treatment of AIDS, development of novel anti-viral strategies is dictated by the medical significance of the AIDS epidemic, side effects of current drugs, and the possible development of drug-resistant HIV strains (Larder and Kemp, 1989; Richman et al., 1991; Moreno et al., 2010). The process of virus assembly, controlled by the HIV-1 Gag proteins, represents an attractive target for such therapies. Findings on the interplay between MA and RNA lay a foundation for determining how HIV-1 MA matrix binds RNA, and the role of MA–RNA interactions in HIV replication. Furthermore, these findings pave the way for efforts to use the MA–RNA interaction as a potential target for a new class of HIV assembly inhibitors. These interactions can be monitored with *in vitro* techniques, making them suitable for screening purposes. Consequently, assays that facilitate the identification of potential inhibitors of MA–RNA interactions have been developed. The reasoning here is that molecules that interfere with the binding of RNAs to MA may impair either an essential MA–RNA binding function, the overlapping MA-PI(4,5)P2 binding function, or both. Based on this, we have designed novel high throughput screens (HTS) in which small-molecule competitors to MA–RNA binding may be identified. The basic assay involves binding of C-terminally His-tagged MyrMA to 96-well nickel-NTA plates, incubation of the plates with biotin-Sel15 RNA in the presence or absence of potential competitors, and colorimetric determination of bound biotin-Sel15 (Alfadhli et al., 2013). Using the MA–RNA binding assay, a library of 14,000 compounds was screened for inhibition of MyrMA–Sel15 RNA binding, looking for candidates that significantly reduced Sel15 RNA binding to MyrMA. The robustness of the assay was indicated by the consistently large difference between samples containing no inhibitor versus those using untagged Sel15 RNA as an inhibitor control, and a favorable Z screening window coefficient (Zhang et al., 1999) of 0.69 for the screen. Using this assay, a small group of compounds that compete with RNA for MA binding was identified. Interestingly, three of the four best inhibitor candidates were thiadiazolanes. These potential inhibitors were characterized with respect to MA binding by NMR, FA, and electrophoretic mobility shift assays (EMSA). Importantly, results showed that MA–thiadiazolanes binding sites do overlap the MA–RNA binding site, validating the concept of such a screening effort. The thiadiazolanes also were shown to inhibit HIV-1 replication in cell culture, but unfortunately also demonstrated cytotoxicity in the 10–20 mM range (Alfadhli et al., 2013). Despite this, such efforts should open the door to the development of new classes of HIV antivirals that target MA and its nucleic-acid-binding pocket.

## Conflict of Interest Statement

The authors declare that the research was conducted in the absence of any commercial or financial relationships that could be construed as a potential conflict of interest.
